# Reproductive Cancer Risk Factors in Women With Myotonic Dystrophy (DM): Survey Data From the US and UK DM Registries

**DOI:** 10.3389/fneur.2019.01071

**Published:** 2019-10-11

**Authors:** Cecilia Higgs, James E. Hilbert, Libby Wood, William B. Martens, Chiara Marini-Bettolo, Nikoletta Nikolenko, Rotana Alsaggaf, Hanns Lochmüller, Richard T. Moxley, Mark H. Greene, Youjin Wang, Shahinaz M. Gadalla

**Affiliations:** ^1^Division of Cancer Epidemiology and Genetics, Clinical Genetics Branch, National Cancer Institute, Bethesda, MD, United States; ^2^Department of Neurology, Neuromuscular Disease Center, University of Rochester Medical Center, Rochester, NY, United States; ^3^John Walton Muscular Dystrophy Research Centre, Institute of Genetic Medicine, Newcastle University, Newcastle upon Tyne, United Kingdom; ^4^National Hospital for Neurology and Neurosurgery, University College London Hospitals NHS Foundation Trust, London, United Kingdom; ^5^Department of Neuropediatrics and Muscle Disorders, Faculty of Medicine, Medical Center, University of Freiburg, Freiburg, Germany; ^6^Centro Nacional de Análisis Genómico (CNAG-CRG), Center for Genomic Regulation Barcelona, Institute of Science and Technology (BIST), Barcelona, Spain; ^7^Children's Hospital of Eastern Ontario Research Institute, University of Ottawa, Ottawa, ON, Canada; ^8^Division of Neurology, Department of Medicine, The Ottawa Hospital, Ottawa, ON, Canada

**Keywords:** myotonic dystrophy, Steinert's disease, female reproductive factors, benign tumor, cancer, endometrial cancer, ovarian cancer

## Abstract

**Introduction:** Recent evidence demonstrates that women with myotonic dystrophy type 1 are at increased risk of reproductive organ tumors. However, studies of reproductive cancer risk factors in those patients are lacking.

**Methods:** Using questionnaires, we collected and analyzed personal history information related to cancer risk factors from women enrolled in a UK and US registry for myotonic dystrophy (*dystrophia myotonica*; DM) patients.

**Results:** The survey was completed by 242 DM type 1 (DM1) and 44 DM type 2 (DM2) women enrolled in the UK Registry (*N* = 124) and the US National Registry (*N* = 162). The mean age at DM1 diagnosis was 33.8 years (standard deviation, *SD* = 13.2) and for DM2 was 49.2 (*SD* = 13.0). Mean age at survey was 48.7 (*SD* = 12.8) and 59.1 years (*SD* = 12.8) for DM1 and DM2, respectively. There were no statistically significant differences between DM1 and DM2 regarding menstrual history or fertility-related factors. Yet, women with DM2 were more likely to have used menopausal hormone therapy (HT) than women with DM1 (52.3 vs. 22.1%, *p* < 0.0001), and more women with DM2 had a hysterectomy (53.5 vs. 29.5%, *p* < 0.01). These differences were not statistically significant after age adjustment (*OR* = 2.00, *p* = 0.08, and *OR* = 1.40, *p* = 0.38, respectively). The frequency of self-reported reproductive organ tumors was not significantly different comparing DM1 to DM2 (*p* = 0.28). However, the data suggested that women with DM2 appear to have a lower risk of malignant tumors compared to those with DM1 (*OR* = 0.72, *p* = 0.69).

**Discussion:** Our study is the first to characterize a wide range of reproductive risk factors in women with DM. We observed no significant differences between DM1 and DM2 in the factors that were evaluated, which suggests that the known excesses of ovarian and endometrial cancer previously reported in women with DM1 cannot be attributed to greater prevalence of standard cancer-related reproductive risk factors. Larger studies evaluating the possible link between reproductive cancer risk factors and risk of tumors in women with DM are needed.

## Introduction

The myotonic dystrophies (*dystrophia myotonica;* DM), are two inherited, multisystem, autosomal dominant disorders that primarily affect skeletal muscle (1). Myotonic dystrophy type 1 (DM1, “Steinert's disease”) is caused by an unstable trinucleotide (CTG) repeat expansion in the 3′-UTR of the dystrophia myotonica-protein kinase (*DMPK*) gene (2–4). Myotonic dystrophy type 2 (DM2) is caused by an unstable tetranucleotide (CCTG) repeat expansion in the CCHC-type zinc finger nucleic acid binding protein (*CNBP*) gene (5, 6). Clinical phenotypes of DM1 and DM2 share many features, including progressive muscle weakness, myotonia, and other organ abnormalities, such as, cardiac conduction defects, gastrointestinal alterations, endocrine disturbances (especially insulin resistance), and characteristic cataracts (7–9).

Recent evidence has shown that DM1 patients have an increased risk of both benign and malignant tumors (10–13), including those of the female reproductive organs. A large population-based study used data from the Swedish and Danish population-based patient registries to show that women with DM have ~5- and 8-fold higher risk of ovarian and endometrial cancer than the general population, respectively (13). Studies of the Basque DM1 cohort (14) and from France and the UK (15, 16) observed similar findings. Cross-sectional studies from Italy and the UK reveal that uterine fibroids are the most commonly reported benign tumor in women with DM1 or DM2 (17, 18).

A better understanding of the medical and family history in DM1 and DM2 patients, combined with current standard clinical assessment of female reproductive factors, may shed light on potential risk factors for these cancers and in turn lead to strategies for prevention and early detection. However, studies with information related to female reproductive cancer risk factors in DM are lacking. In this study, we report a wide range of reproductive factors in women with DM1 or DM2, focusing on risk of cancers of the female reproductive system, using data collected from patients enrolled in DM registries from the US and UK.

## Materials and Methods

### Study Population and Data Collection

This study included 286 women, genetically and/or clinically confirmed as having DM and self-enrolled in the UK DM Registry (19) (https://www.dm-registry.org/uk/; *N* = 124: DM1 = 120, DM2 = 4) or the US National Registry of DM and Facioscapulohumeral Muscular Dystrophy (20) (US DM registry; https://www.urmc.rochester.edu/neurology/national-registry.aspx; *N* = 162: DM1 = 122, DM2 = 40), who responded to the reproductive history questionnaire. DM diagnosis was confirmed by the patient-designated healthcare professional in the UK and medical record review in the US (19, 20).

Details describing the methodology of data collection and information obtained have been published previously (18, 21, 22). Briefly, a self-administered questionnaire was sent to DM patients enrolled in the US (*via* mail) or UK (primarily *via* email, followed by mail for non-responders) DM registries. The questionnaire requested information on personal history of benign and malignant tumors, familial history of cancer, and known cancer risk factors, including reproductive history. Baseline demographic and DM clinical factor data were obtained from relevant registry databases. Research activities of the US DM registry were approved by the University of Rochester's Institutional Review Board, and those of the UK DM Registry were approved by the UK Research Ethics Service. This report is part of our comprehensive effort aimed at more detailed characterization of the recently-recognized cancer susceptibility in DM patients (18, 21–23).

### Collection of Female Reproductive Cancer Risk Factor Information

The questionnaire collected the following information: age at menarche, birth control pill use and duration, consultation for infertility, pregnancy history (number of pregnancies and live-born children), time of last regular menstrual period, menopausal hormone therapy (HT) use and duration, and history of hysterectomy. Information on history of gynecologic tumors was determined from the patient's response to the following questions: “Have you ever been diagnosed with any benign (non-cancerous) tumor?” and “Have you ever been diagnosed with any type of cancer (a malignant growth or invasive tumor)?” If patients responded “yes,” they were asked to specify the tumor type/site and date- or age-at-diagnosis.

### Statistical Analysis

We used chi-square or Fisher's exact test to compare the frequency of categorical variables between DM1 and DM2 patients, and *t*-test for continuous variables. To minimize the possible impact of confounding by age, we used logistic regression models to adjust all comparisons for age at survey. All analyses were conducted using SPSS Version 25.0 (IBM Corp. Armonk, NY) with statistical significance defined as two-sided *p* < 0.05.

## Results

### Characteristics of Female DM Patients Participating in the Study

DM patients enrolled from the US DM registry were older at survey and at DM diagnosis (Table 1). In both countries, DM2 patients were older than DM1 patients (mean age at survey = 59.8 vs. 51.2, *p* = 0.0001 in the US; 52.2 vs. 46.2, *p* = 0.37 in the UK). As expected, DM2 patients were diagnosed at an older age (mean age at diagnosis = 49.7 vs. 33.7 in the US; 42.3 vs. 33.8 in the UK), and were older at first DM2 symptom than those with DM1 (mean age at first DM symptom = 35.9 vs. 26.8 years in the US; 41.0 vs. 27.8 years in the UK).

**Table 1:** Characteristics of female DM patients in the US and UK registries.

	**US (*****n*** **=** **162)**		**UK (*****n*** **=** **124)**		
**Patient characteristics**	***n***	**Mean (SD)**	***n***	**Mean (SD)**	***P*-value^a^**
Age, years					
At survey	162	53.3 (12.6)	124	46.4 (13.2)	<0.0001
At DM diagnosis	161	37.6 (15.0)	120	34.0 (12.9)	0.04
At first DM symptom	156	29.0 (15.0)	112	28.0 (13.7)	0.60
	***n***	**%**	***n***	**%**	
DM subtype					<0.0001
DM1	122	75.3	120	96.8	
DM2	40	24.7	4	3.2	
Race					0.20^b^
White	154	95.1	121	98.4	
Other	8	4.9	2	1.6	

*DM, myotonic dystrophy; SD, standard deviation*.

a *t-test for continuous variables and chi-square test for categorical variables unless otherwise specified*.

b *Fisher's exact test*.

### Comparison of Female Reproductive Factors Between DM1 and DM2 Patients

Table 2 summarizes female reproductive factors in patients with DM1 and DM2. The median age at menarche was 13 years for both DM1 and DM2 patients (DM1 range = 9–23, DM2 range = 9–16). DM1 and DM2 patients reported similar use of oral contraceptives, a higher frequency of self-reported menopausal HT use in patients with DM2 was noted (52.3 vs. 22.1%, in DM2 and DM1, respectively, *p* < 0.0001). Hysterectomy was reported by 33.3% of the patients (*n* = 89 among 267 responders); 56 of whom also had oophorectomy. Approximately half of those with hysterectomy (48.3%, *N* = 43) reported a history of gynecologic tumor (DM1 patients = 32, 74.4%, DM2 patients = 11, 25.6%). Surgical menopause (hysterectomy and/or oophorectomy) was the most common reason for cessation of menses (≥6 months) in both DM1 and DM2.

**Table 2:** Selected female reproductive cancer risk factors among women with DM by disease subtype.

	**DM1 (*****n*** **=** **242)**			**DM2 (*****n*** **=** **44)**		
**Female reproductive factors**	***N responded***	**Mean (SD) or *N* (%)**		***N responded***	**Mean (SD) or *N* (%)**	***P*-value^a^**
Birth control pill use	167			36		0.60^b^
Ever		145 (86.8%)			30 (83.3%)	
Never		22 (13.2%)			6 (16.7%)	
Years on birth control pill	116	8.98 (7.9)		29	7.79 (7.6)	0.47
Fertility-related consultation	152			35		0.36
Ever		56 (36.8%)			10 (28.6%)	
Never		96 (63.2%)			25 (71.4%)	
Pregnancy	225			42		0.34
Ever		149 (66.2%)			31 (73.8%)	
Never		76 (33.8%)			11 (26.2%)	
Number of live births; mean (SD)	126	1.95 (0.9)		29	2.31 (1.2)	0.15^c^
1		44 (34.9%)			8 (27.6%)	0.44^c^
2–3		70 (55.6%)			16 (55.2%)	
≥4		12 (9.5%)			5 (17.2%)	
Reason no menstrual period ≥6 months	141			37		0.05
Natural menopause as the reason		46 (32.6%)			16 (43.2%)	
Surgery to remove uterus or ovaries		62 (44.0%)			19 (51.4%)	
Other reasons		33 (23.4%)			2 (5.4%)	
Menopausal HT use	213			44		<0.0001
Ever		47 (22.1%)			23 (52.3%)	
Never		166 (77.9%)			21 (47.7%)	
Years on menopausal HT	42	8.19 (9.1)		22	7.43 (8.4)	0.75
Hysterectomy^d^	224			43		<0.01
Ever		66 (29.5%)			23 (53.5%)	
Never		158 (70.5%)			20 (46.5%)	
Gynecologic tumor history	232			43		0.28
Benign only^e^		50 (21.6%)			14 (32.6%)	
Malignant ± benign^e^		10 (4.3%)			2 (4.6%)	
No gynecologic tumor history		172 (74.1%)			27 (62.8%)	

*DM, myotonic dystrophy; SD, standard deviation*.

a *t-test for continuous variables and chi-square test for categorical variables unless otherwise specified*.

b *Fisher's exact test*.

c *p-value comparing number of live births = 0.15; p-value comparing frequencies of 1, 2–3, ≥4 live births between DM1 and DM2 = 0.44*.

d *Among N = 89 women who were hysterectomized, N = 56 women also reported a history of oophorectomy*.

e *Benign only (n = 69 patients): uterine fibroid (n = 46), ovarian cysts (n = 33), uterine fibroid + ovarian cysts (n = 12), other gynecologic benign tumor (n = 4); malignant only (n = 7 patients): endometrial or uterine cancer (n = 3), ovarian cancer (n = 3), cervical cancer (n = 1); benign + malignant (n = 5 patients): ovarian cysts + cervical cancer (n = 1), ovarian cysts + endometrial or uterine cancer (n = 1), ovarian cysts + ovarian cancer (n = 1), uterine fibroids + ovarian cancer (n = 1), uterine fibroids, ovarian cysts + ovarian cancer (n = 1)*.

Gynecological tumors were reported by more than one-fourth of the patients. DM1 patients reported 64 benign (in 54 patients), and 10 malignant gynecological tumors (in 10 patients). DM2 patients reported 19 benign (in 15 patients) and 2 malignant gynecological tumors (in 2 patients). Overall, uterine fibroids were the most frequent benign tumor (*N* = 46 patients, prevalence = 16.7%), followed by ovarian cysts (*N* = 33 patients, prevalence = 12.0%). For malignant tumor, 6 women (2.2%) reported ovarian cancer, 4 reported endometrial or uterine cancer (1.4%), and 2 reported cervical cancer (0.7%). Among the 12 women with a gynecologic cancer, 5 reported that they also had a benign gynecological tumor.

In age-adjusted analyses, no statistically significant differences between DM1 and DM2 were noted in any of the factors analyzed (Table 3). However, the data suggested that DM2 patients may be more likely to have undergone hysterectomy (*OR* = 1.40, *p* = 0.38) or receive menopausal HT (*OR* = 2.00, *p* = 0.08), and less likely to have infertility-related consultations (*OR* = 0.74, *p* = 0.49), or malignant tumors of the reproductive organs (*OR* = 0.72, *p* = 0.69).

**Table 3:** Age-adjusted comparison of key female reproductive cancer risk factors between patients with DM2 vs. DM1.

**Female reproductive factors**	**Adjusted OR**	**95% CI**	***P*-value**
Ever use of birth control pill	1.20	(0.42–3.43)	0.73
Infertility-related consultation	0.74	(0.32–1.72)	0.49
Ever been pregnant	0.78	(0.34–1.77)	0.55
Ever used menopausal HT	2.00	(0.93–4.31)	0.08
Hysterectomy^a^	1.40	(0.66–2.98)	0.38
Gynecologic tumor history^b^			
Benign tumor only	1.15	(0.53–2.49)	0.72
Malignant cancer	0.72	(0.14–3.72)	0.69

*DM1, myotonic dystrophy type1; DM2, myotonic dystrophy type2; OR, odds ratio; CI, confidence interval*.

a *Among N = 267 who responded to “hysterectomy ever”. Reference: women who never had hysterectomy*.

b *Among N = 275 who responded to the cancer and tumor history questionnaires. Reference: women with no gynecologic tumor history*.

## Discussion

In this cross-sectional study of 286 women with DM1 or DM2, we explored differences in reproductive cancer risk factors by DM subtype. Age-adjusted analyses showed no statistically significant differences; however, the data suggested that DM2 patients may be more likely to have undergone hysterectomy and to have used menopausal HT.

In this study, we observed similar ages at menarche for women with DM1 or DM2 (median = 13 years). This was similar to age at menarche reported in the US general population based on data from 2,510 children and adolescents of non-Hispanic whites, blacks, and Mexican Americans in the third National Health and Nutrition Examination Survey (median = 12.4 years) (24). For age at menopause, we did not directly collect this information. However, most patients (DM1 = 91.3%, DM2 = 100%) who reported natural menopause were >50 years old at survey. The median age of natural menopause in the US is 52.6 years (25). On the other hand, 81 patients reported surgical menopause, 21 of which (26%) occurred at or before age 50. One-third of the DM patients in the current study reported a history of hysterectomy. The US prevalence for women 15–44 years of age is 4.0% (26, 27). In the UK, the estimated prevalence of hysterectomy for all ages is approximately 9%, with a peak increase at age 55 and older that reaches 20% (28). Similar to previous studies in DM (17, 18), uterine fibroids were common, affecting 16.7% of the DM patients, followed by ovarian cysts, affecting 12%. The prevalence of uterine fibroids noted in this study is higher than that reported in the general population from the US (6.9%) and UK (4.5%) in a large study of 21,746 women aged 15–49 years from eight countries (29).

The molecular basis underlying tumor development in the female reproductive systems among DM patients has yet to be identified. Ongoing studies seek a better understanding of whether DM disease severity correlates with tumor development. Alsaggaf et al. have provided the first data suggesting that DM1-related cancer susceptibility may be positively correlated with disease severity in adult onset DM1 (16). Upregulation of *Wnt-*β*-catenin* signaling (30) and loss of *DMPK* heterozygosity have been hypothesized as etiologically important (31). Interestingly, a recent gene expression analysis showed microRNA-200c and microRNA-141 tumor suppressor gene expression is sex-dependent in DM1, in which significant downregulation was observed among female DM1 patients (14). Downregulation of miRNA-200c and miRNA141 were implicated in uterine fibroid (32) and endometriosis (33) pathogenesis, respectively. Additionally, dysregulation of miR-200 family has been associated with ovarian cancer (34) and endometrial cancer development (35).

Our current investigation is the first study to characterize a wide range of reproductive risk factors in women with DM. The strengths of our current study include its relatively large sample size, representation of DM patients from two countries, and DM diagnosis having been validated by medical professionals. Our investigation is limited by the self-reported nature of the collected information. Since the study analyzed data from DM patients who were self-enrolled in the DM registries, the results may not be generalizable to the entire DM population. However, our study population did have similar distribution of age-at-diagnosis to the DM general population. Our study focused on DM patients and lacked matched control comparisons, so we used published general population estimates which may not be age comparable to our study population. Our questionnaire lacked information on birth control methods other than pills such as injections. Due to the small number of reported cancers in study participants, we were not able to formally test associations between reproductive factors and risk of specific neoplasms. Also, our reported frequencies may be overestimated in situations of missing information due to patient non-response since denominators were only based on the number of responders.

In conclusion, our study found no significant reproductive cancer factor differences between female DM1 and DM2 subjects, except for a possibility of a higher frequency of hysterectomy and HT use in DM2 patients. The older age of the DM2 patient cohort may have contributed to these observed differences. The observed similarities between DM1 and DM2 cohorts regarding menstrual history or fertility-related factors suggest that the known excesses of ovarian and endometrial cancer seen in DM1 subjects cannot be attributed to greater prevalence of standard gynecologic cancer risk factors. In the future, larger studies evaluating the possible link between reproductive cancer risk factors and risk of tumors in women with DM are needed.

## Data Availability Statement

The datasets generated for this study are available on request to the corresponding author.

## Ethics Statement

This study was reviewed and approved by the University of Rochester's Institutional Review Board and activities of the UK DM registry were approved by the UK Research Ethics Service. The patients/participants provided their written informed consent to participate in this study.

## Author Contributions

SG and MG contributed to the project conceptual design. JH, LW, WM, CM-B, HL, and RM contributed to the data collection and management. CH, YW, and SG contributed to the statistical analysis, data interpretation, and manuscript draft. All authors contributed to the manuscript critical review and approval.

### Conflict of Interest

The authors declare that the research was conducted in the absence of any commercial or financial relationships that could be construed as a potential conflict of interest.

## Abbreviations

CNBP: CCHC-type zinc finger nucleic acid binding protein
CTG: Trinucleotide
CCTG: Tetranucleotide
CI: Confidence interval
DM: Myotonic dystrophy
DM1: Myotonic dystrophy type 1
DM2: Myotonic dystrophy type 2
*DMPK*: dystrophia myotonica-protein kinase
FSHD: US National Registry of DM and Facioscapulohumeral Muscular Dystrophy
HT: Hormone therapy
OR: Odds ratio
SD: Standard deviation
UF: Uterine fibroid.
